# Utilization of basic diabetes mellitus services among adult patients with diabetes mellitus at Mbarara Regional Referral Hospital diabetes clinic, Uganda: a cross-sectional study

**DOI:** 10.3389/fcdhc.2024.1290559

**Published:** 2024-12-03

**Authors:** Dan Muramuzi, Richard Mugambe, Laban Muteebwa, Ipola Patrick Albert, Lawrence Nduhukyire, Claire Nnannyanzi, Aggrey David Mukose

**Affiliations:** ^1^ Department of Epidemiology and Biostatistics, School of Public Health, College of Health Sciences, Makerere University, Kampala, Uganda; ^2^ Department of Disease Control and Environmental Health, School of Public Health, College of Health Sciences, Makerere University, Kampala, Uganda; ^3^ Clinical Epidemiology Unit, School of Medicine, College of Health Sciences, Makerere University, Kampala, Uganda

**Keywords:** diabetes mellitus, utilization, basic diabetic care, Mbarara Regional Referral Hospital, adult patients

## Abstract

**Background:**

Utilization of diabetic care services remains a challenge among adult patients in low- and middle-income countries. Optimal utilization of diabetic care services could reduce morbidity and mortality and delay the development of long-term complications. This study assessed the utilization of basic diabetic care services and associated factors among diabetes mellitus patients at Mbarara Regional Referral Hospital in Western Uganda.

**Methods:**

We enrolled 208 patients with either type 1 or 2 diabetes mellitus in a cross-sectional study between June and August 2022 at Mbarara Regional Referral Hospital, Uganda. Consecutive sampling was used to select patients with diabetes mellitus who attended the diabetes clinic based on their scheduled date of appointment. Optimal utilization of basic diabetic services was defined as receiving at least three of the four core services: health education, assessment of vital signs (blood sugar, blood pressure, and body mass index), assessment of diabetic complications, and diabetic drug refills. Modified Poisson regression analysis was used to assess the determinants of utilization of basic diabetic services in STATA version 14.0.

**Results:**

Three-quarters of the study participants were female patients (75.0%), and the median (inter-quartile range, IQR) age was 52 (43, 56). Moreover, 62.0% [95% confidence interval (CI): 55.3–68.7] self-reported to have utilized basic diabetic care services. In the adjusted analysis, waiting longer than 120 min to receive any service [adjusted prevalence ratio (aPR) 0.46, 95% CI 0.27–0.80), being 45–54 years old (aPR 1.48, 95% CI:1.11–1.98), and being 55–60 years old (aPR, 1.38, 95% CI: 1.02 1.86)] were significantly associated with the utilization of basic diabetic care services.

**Conclusion:**

Utilization of basic diabetic care services among adult patients remains sub-optimal. Age and waiting time were significantly associated with the utilization of diabetic care services. Interventions should be directed toward shortening the waiting time in service delivery at diabetes clinics.

## Introduction

Globally, the prevalence of diabetes mellitus (DM) has risen from 8.3% in 2013 to 9.3% in 2019, and this is expected to rise further by 2045 (1, 2), with an estimated 629 million people by 2045 in low- and middle-income countries (LMIC) (3). It is estimated that at least 3%–6% of the world`s diabetic population live in Africa (4, 5). There is a rapid increase in the prevalence of DM among the adult population in sub-Saharan Africa (SSA) countries with varying rates of 2.0% in Gambia, 6.3% in the Congo, 9.3% in South Africa, and as high as 14.8% in Mauritius (6) and 1.4%–3% in Uganda (2, 7). In Uganda, the Western region has the highest prevalence of DM, second to the Central region (2). A recent study at Mbarara Regional Referral Hospital (MRRH) reported a high prevalence of poor glycemic control among diabetic patients (84.3%), which is partly due to poor utilization of diabetic services (8).

According to the Uganda Ministry of Health (MoH), basic DM care involves health education, assessment of vital signs [blood pressure, body mass index (BMI), and blood sugar], diabetic drug refill, and assessment of complications (for example, neuropathy, retinopathy, and diabetic foot). Optimal utilization of basic diabetic services is cardinal to monitor blood sugar, diabetic treatment, and diabetic-related complications (9, 10). However, the MoH reported that the outpatient attendance of DM patients was very low (0.5%) in 2019/2020 (11), which suggests sub-optimal utilization of healthcare services (12). Sub-optimal utilization of basic diabetic services is associated with the development of poor health outcomes (13).

Several individual and health system factors have been reported to be associated with sub-optimal utilization of diabetic services—for example, limited operating hours at health facilities, frequent stockouts of essential drugs, high costs of medicines, and poor quality of drugs have been reported to influence the utilization of the services (14). At Mbarara Regional Referral Hospital (MRRH), efforts to improve diabetic service utilization include a voluntary patient association that provides peer support and help in the cost-sharing of some diabetic treatments (15).

Despite the above-mentioned interventions, utilization of basic diabetic care services remains a challenge among diabetic care patients partly due to other socioeconomic, health system, and environmental factors (16). There are reports of poor glycemic control in the study setting (8), and long waiting times and understaffing are among the most common challenges (17). Therefore, this study sought to assess the utilization of basic diabetic services and associated factors among adult patients with diabetes at MRRH.

## Materials and methods

This was a cross-sessional study conducted at a diabetes clinic at Mbarara Regional Referral Hospital (MRRH) located in Western Uganda. MRRH is a public secondary-level hospital with a bed capacity of approximately 350 beds and serves as a teaching hospital for Mbarara University of Science and Technology (MUST). The diabetes clinic offers specialized care tailored to the unique needs of individuals living with diabetes. Unlike other medical clinics, this clinic has a multidisciplinary team comprised of physicians, trained diabetes educators, nurses, and other healthcare professionals with extensive expertise in diabetes management, with an estimated patient number of over 1,000 by 2020. The hospital serves approximately 12 districts in Western Uganda, an area characterized by subsistence farmers. The community also has people who operate in mobile markets to earn a living, which ultimately interferes with health-seeking patterns. The clinic is one of the walk-in clinics that operate once a week—every Thursday—and is managed by a medical officer and two nurses, and the patients are provided with scheduled appointments with an interval of 2 to 3 months.

### Study population and eligibility criteria

We conducted the study among adult diabetic patients with either type 1 or type 2 diabetes mellitus, aged 35 years and above and seeking care at the MRRH diabetes clinic between June and August 2022. The age of 35 years and above was chosen due to the reported high prevalence of diabetes among this sub-population (18). We enrolled the participants based on their scheduled appointments, and in case of multiple visits, the study team only considered the most recent visit in the last 90 days preceding data collection. The diabetic patients at this clinic are given appointment dates for the subsequent visit for routine diabetic care (usually after 3 months). This is often for patients whose blood sugar levels have been controlled; however, patients are advised to return to the clinic if they experience diabetes-related challenges before the scheduled dates. The participants were eligible if they were aged 35 years and above and gave written informed consent to participate in the study. Diabetic patients who were very ill were excluded from the study.

### Study variables

The dependent variable was utilization of basic diabetic care services. This was defined as the proportion of participants who utilized at least three basic diabetic care services by self-report in the 90 days preceding data collection. Measurement of the outcome included four questions (1): Did you receive health education about diabetic care at this facility? (2) Did you get all of the following—blood pressure, BMI, and blood sugar—assessed while at the facility? (3) Did you have any diabetic complications assessed (diabetic foot, blurring of vision, and peripheral nerve damages)? (4) Did you refill your diabetic drugs at this facility? The independent variables included age, gender, marital status, religion, place of residence and occupation, presence of diabetic complications status, knowledge about diabetic disease, education level, living arrangements, reminder strategies, distance to the facility, time to reach the facility, waiting time at the facility, and self-blood glucose monitoring. Knowledge about diabetes was assessed using four questions which assessed the participant’s knowledge on diabetes risk factors, prevention of diabetes mellitus, blood sugar control, and diabetic-related complications by providing examples. The response to each question was recorded as “yes” if the participant mentioned at least three correct responses for each of the four questions assessing knowledge. The response would be scored “no” if the participant could not mention at least three correct responses on examples of diabetes risk factors, ways to prevent diabetes mellitus, ways to control blood sugar, and examples of diabetic-related complications.

### Sampling procedure

The prospective study participants were found at the diabetes clinic in the waiting area during their visit. Consecutive sampling was used to recruit patients who were attending the diabetes clinic during the study period until the desired sample size was achieved.

### Data collection procedure

A structured pretested questionnaire was used to collect data from the patients. This tool was adapted from a study conducted in Bangladesh on the utilization of diabetic care services (19). The questionnaire was translated into Runyankole, which was the commonly spoken language in the study area.

### Data analysis

The data was analyzed in STATA version 14.0 (TX, USA). For descriptive statistics, categorical variables were summarized using frequencies and percentages, while continuous variables were summarized using median and *r* interquartile range. The proportion of utilization of basic diabetic services was measured as the number of diabetic patients that utilized basic diabetic services out of the total number of patients, and its logit CI was presented. The relationship between utilization of basic diabetic services and independent variables was assessed using the modified Poisson regression with robust standard errors. At unadjusted analysis, the crude prevalence ratios (cPR), their 95% confidence interval (CI), and *P*-values were reported. Independent variables with a *P*-value of <0.2 were considered in the adjusted analysis. At adjusted analysis, model building followed a backward elimination procedure. Interaction was assessed using a chunk test, whereas confounding was assessed using a cutoff of 10%. An independent variable that influenced the aPR of another independent variable in the model by a magnitude of >10% was regarded as a confounder. The independent variables in the adjusted model with a *P*-value <0.05 were regarded as statistically significant. The aPR, its 95% CI, and the *P*-values were reported.

## Results

### Socio-demographic characteristics of study participants

Three quarters of the study participants were women (75% (156/208). The median (IQR) monthly family expenditure on diabetes drugs was US$ 51.95 (US$ 25.97–77.92), over a third of the patients (36.5%, 76/208) were aged 45–54 years, 69.2% (144/208) were living in rural areas, and over a third of the patients (38.5%, 80/208) had no formal education. Slightly over a third of the patients (40.4%, 84/208) were Catholics, and almost two-thirds of the patients (63.9%, 133/208) were unemployed. Only 8.2% (17/208) had formal employment, and about three quarters (73.1%) of the patients were married. A majority of the patients (70.2%, 146/208) had inadequate knowledge on the risk factors of diabetes mellitus, and nearly two-thirds (60.1%, 125/208) of the patients had inadequate knowledge on how to prevent diabetes (**Table 1**).

**Table 1:** Individual characteristics of 208 diabetic patients who participated in the study at Mbarara Regional Referral Hospital.

Study variables Categories		Frequencies (*n* = 208)	Percentage (%)
Age in complete years, median (IQR) 52 (43, 56)			
Sex	Male	52	25.0
	Female	156	75.0
Religion	Anglican	82	39.5
	Catholic	84	40.4
	Moslem	31	14.9
	Born again	11	5.3
Marital status	Never married	5	2.4
	Married	152	73.1
	Widow/widower	28	13.5
	Separated/divorced	23	11.1
Highest level of education attained	No formal education	80	38.5
	Primary	78	37.5
	Secondary	28	13.5
	Advanced level	6	2.9
	College/university	16	7.7
Occupation of participants	Unemployed	133	63.9
	Self-employed	58	27.9
	Formal employment	17	8.2
Number of biological children	1–6	135	64.9
	7–10	60	28.9
	11 and above	13	6.3
Area of residence	Urban	64	30.8
	Rural	144	69.2
Knowledge on risk factors to developing DM	Yes	62	29.8
	No	146	70.2
Knowledge on DM prevention	Yes	83	39.9
	No	125	60.1
Knowledge on control of DM	Yes	179	86.1
	No	29	13.9
Knowledge about complications	Yes	175	84.1
	No	33	15.9
Perception of participants on DM	Negative perception	106	51.0
	Positive perception	102	49.0
Seeking care from other facility	No	127	62.0
	Yes	79	38.0

### Utilization of basic diabetic care services

Almost two-thirds of the study participants (62.0%, 95% CI: 55.3–68.7) utilized the basic diabetic care services.

### Factors associated with the utilization of basic diabetic care services among adult patients

In the adjusted analysis, waiting longer than 120 min to receive any service (aPR 0.46, 95% CI: 0.27–0.80) was significantly associated with lower utilization of basic diabetic care services, whereas being 45–54 years old (aPR 1.48, 95% CI: 1.11–1.98) and 55–60 years old (aPR, 1.38, 95% CI: 1.02–1.86) was significantly associated with higher utilization of basic diabetic care services (**Table 2**).

**Table 2:** Bivariate and multivariate analysis for factors associated with the utilization of basic diabetic care services among 208 adult patients with diabetes mellitus at Mbarara Regional Referral Hospital.

Variables		Utilization of service			cPR		95% CI	*P*-value	aPR	95% CI	*P*-value
		No, *n* (%)	Yes, *n* (%)								
Age											
35–44		35 (53.9)	30 (46.2)		Ref				Ref		
45–54		22 (28.9)	54 (71.1)		1.54		1.140–2.077	0.005	1.48	1.11–1.98	0.008
55–60		22 (32.8)	45 (67.2)		1.46		1.065–1.988	0.018	1.38	1.02–1.86	0.037
Sex											
Male		18 (34.6)	34 (65.4)		Ref						
Female		61 (39.1)	95 (60.1)		0.93		0.736–1.178	0.550			
Religion											
Anglican		33 (40.2)	49 (59.8)		Ref						
Catholic		30 (35.7)	54 (64.3)		1.08		0.846–1.367	0.550			
Moslem		14 (45.2)	17 (54.9)		0.92		0.636–1.324	0.650			
Born again		2 (18.2)	9 (81.8)		1.37		0.983–1.907	0.050			
Marital status											
Never married		3 (60.0)	2 (40.0)		Ref						
Married		57 (37.5)	95 (62.5)		1.57		0.528–4.616	0.420			
Widow		12 (42.9)	16 (57.1)		1.42		0.465–4.392	0.530			
Separated		7 (30.4)	16 (69.6)		1.74		0.573–5.275	0.330			
Education											
No education		25 (31.3)	55 (68.8)		Ref						
Primary		37 (47.4)	41 (52.6)		0.76		0.590–0.989	0.040			
Secondary		12 (35.3)	22 (64.7		0.94		0.705–1.257	0.680			
Tertiary		5 (31.3)	11 (68.8)		1.0		0.696–1.437	1.000			
Occupation											
Unemployed		40 (30.1)	93 (69.9)		Ref						
Self-employed		32 (55.2)	26 (44.8)		0.64		0.472–0.872	0.005			
Formal employment		7 (41.2)	10 (58.8)		0.84		0.556–1.273	0.413			
Household members											
1–6		56 (41.5)	79 (58.5)		Ref						
7–10		20 (33.3)	40 (66.7)		1.14		0.906–1.432	0.270			
11–15		3 (23.1)	10 (76.9)		1.31		0.944–1.820	0.110			
Area of residence											
Urban		21 (32.8)	43 (67.2)		Ref						
Rural		58 (40.3)	86 (59.7)		0.89		0.714–1.105	0.290			
Knowledge about diabetes											
No		18 (62.1)	11 (37.9)		Ref						
Yes		61 (34.1)	118 (65.9)		1.74		1.077–2.804	0.024			
Perception about diabetes											
Negative		41 (38.7)	65 (61.3)		Ref						
Positive		38 (37.3)	64 (62.8)		1.02		0.826–1.26	0.830			
Means of transport											
By foot		4 (57.1)	3 (42.9)		Ref						
Public means		71 (37.2)	120 (62.8)		1.47		0.617–3.480	0.380			
Private means		4 (40.0)	6 (60.0)		1.4		0.517–3.79	0.510			
Instructions on the use of drugs											
Often times		5 (71.4)	2 (28.6)		Ref						
Always		74 (32.8)	127 (63.8)		2.22		0.680–7.189	0.187			
Waiting time											
30 min		21 (27.6)	55 (72.4)		Ref				Ref		
31–60 min		29 (33.7)	57 (66.3)		0.92		0.745–1.124	0.402	0.90	0.73, 1.10	0.290
61–120 min		8 (50.0)	8 (50.0)		0.69		0.414–1.151	0.156	0.63	0.39, 1.04	0.072
Above 120 min		21 (70.0)	9 (30.0)		0.41		0.235–0.730	0.002	0.46	0.27, 0.80	0.006
Reminders for clinic review											
Always given		1 1 (34.4)	21 (65.4)		Ref						
Never		67 (38.3)	108 (61.7)		0.94		0.71–1.20	0.670			
Distance to facility											
0–25 km		57 (41.0)	82 (59.0)		Ref						
Above 25 km		22 (31.9)	47 (68.1)		1.15		0.92–1.42	0.20			

## Discussion

This study assessed the utilization of basic diabetic care services among adult patients at a diabetes clinic of a secondary-level facility in Uganda. This study revealed that 62% of adult patients with diabetes mellitus utilized basic diabetic care services at the diabetes clinic in the 90 days preceding data collection. While this utilization of 62% is higher than in some settings, it remains sub-optimal for the effective management of diabetes mellitus disease. Similar studies conducted in sub-Saharan Africa have reported a high level of utilization of diabetic services among this patient group—for instance, a study conducted in Cameroon, Mali, Tanzania, and South Africa indicated that patients with diabetes utilized inpatient and outpatient services higher than their counterparts without diabetes mellitus (20). Furthermore, in a cross-sectional study conducted in Bangladesh among adult patients with diabetes mellitus, only 37% checked their blood sugar levels at least once in 3 months, which was used as a measure of utilization of services (21). The observed prevalence in this study could be attributed to improved patient knowledge of diabetic management and readily available services at the clinic.

Patient’s age and longer waiting time to receive any service were significantly associated with higher and lower utilization of basic diabetic care services, respectively, at Mbarara Regional Referral Hospital. These findings are similar to a study done in South Africa, where results indicated that the prevalence of utilization among patients aged above 40 years was slightly high (22). Despite the data mentioned above, according to a study conducted in Ghana, age was not among the factors associated with the utilization of diabetic care services (23). A systematic review (13) also indicated that young adults (18–30 years) were instead associated with poor utilization. The findings from the current study may be due to availability of time and social and financial support from their family members because patients who are aged above 45 years are less likely to be much involved in economic work and therefore can easily honor clinic appointments as scheduled. It should be noted that this study did not assess the financial status of the household members which would otherwise provide some context at the family level. In addition, patients who waited at the clinic for more than 120 min had a lower prevalence of utilizing diabetic care services. This is similar to a study conducted in Canada which indicated that individuals from lower socio-economic groups have lower levels of utilization of healthcare reflected in longer waiting times and fewer referrals for specialist care (24). A study conducted in Omani further indicated that delays in the provision of laboratory results and long waiting times to see the doctor affected diabetic service delivery (25). Similar findings on long waiting times from other studies equally affirmed the findings from this study (26, 27).

### Limitations of the study

This study excluded patients who were severely ill during data collection, yet this could have been caused by poor utilization of diabetic care services. Therefore, this could have introduced selection bias. This study was conducted in a hospital setting for which the patients could have good health-seeking behaviors and thus would be more likely to utilize the basic diabetic care services, and this could have overestimated the outcome.

## Conclusion and recommendation

The results indicated that only 62% of patients with diabetes mellitus utilized basic care services with age and waiting time to receive any service among the significant factors. Given that the clinic has one full-time medical doctor and two nurses, the clinic can utilize task-shifting and team-based care such that nurses can directly get involved in the management of stable diabetic patients who may only need less advanced care. This can help offload some of the workload from physicians, allowing them to focus on more complex cases or patients with acute needs. The clinic should also strengthen patient education and self-management strategies as better ways to promote a positive perception of diabetes mellitus as demonstrated in this study: 51% of patients had a negative perception of diabetes, 15.9% did not have adequate knowledge of complications, 13.9% did not have adequate knowledge on control, and 70.2% did not have adequate knowledge on the risk factors for developing diabetes disease. This could potentially reduce the frequency of clinic visits for routine follow-ups and monitoring, thereby reducing the overall patient load and wait times.

## Data availability statement

The original contributions presented in the study are included in the article/supplementary material. Further inquiries can be directed to the corresponding author.

## Ethics statement

The study was approved by Makerere University School of Public Health, Research and Ethics Committee (MakSPH-REC 034). The study was conducted following the local legislation and institutional requirements. The participants provided their written informed consent to participate in this study.

## Author contributions

DM: Writing – original draft. AM: Supervision, Writing – review & editing. RM: Supervision, Writing – review & editing. LM: Conceptualization, Writing – review & editing. IA: Validation, Writing – review & editing. LN: Writing – review & editing. CN: Writing – review & editing.
